# Early Recognition of Overweight Hyperglycaemia May Improve Clinical Outcomes in Type 2 Diabetes

**DOI:** 10.17925/EE.2023.19.1.33

**Published:** 2023-03-27

**Authors:** Anand Chockalingam, Pandiyan Natarajan, Smrita Dorairajan, Uzma Khan

**Affiliations:** 1. Division of Cardiovascular Medicine, University of Missouri, Columbia, MO, USA; 2. Cardiology Section, Harry S Truman VA Medical Center, Columbia, MO, USA; 3. Nova IVF Fertility, Chettinad Super Speciality Hospital (Retired), Chennai, India; 4. Nephrology Section, Harry S Truman VA Medical Center, Columbia, MO, USA; 5. Division of Endocrinology, University of Missouri, Columbia, MO, USA

**Keywords:** Diabetes, hyperglycaemia, insulin resistance, lifestyle, overweight hyperglycaemia, severe obesity, type 2 diabetes

## Abstract

Diabetes is the ninth leading cause of death, directly accounting for 1.5 million deaths annually worldwide. Despite several breakthrough discoveries, little progress has been made in type 2 diabetes outcomes over the past 100 years. Younger age (below 60 years), a diet high in calories and processed food, and severe obesity (body mass index >35 kg/m^2^) may identify reversible beta cell dysfunction. Much of the clinical presentation pertains to flooding the body’s adaptive limits with overnutrition. Recognizing this as a global societal trend brought about by lifestyle changes, sedentary work, mental stress and unlimited access to calorie-dense foods is crucial. Insulin resistance and genetic abnormalities cannot account for the dramatic increase in diabetes, from only 1% five decades ago to nearly 10% today. Obesity – and not insulin resistance – is at the core of the problem. As well as hyperglycaemia, end-organ damage can also be reversed with diet and weight loss in many affected individuals. We present the evolution of our understanding and compelling reasons to reframe diabetes in the severely obese to what it really is – overweight hyperglycaemia. This may shift societal perception, governmental funding, workplace reformations and individual engagement with healthy lifestyles. The objective of this review is to better understand global trends and the potential to improve outcomes by reframing the diabetes narrative towards remission. This may shift societal perception, governmental funding, workplace reformations and individual engagement with healthy lifestyles.

Diabetes affects an estimated 537 million adults and was responsible for 6.7 million deaths in 2021, worldwide.^1^ The International Diabetes Federation estimates that 3 out of 4 adults with diabetes live in low-and middle-i ncome countries.^1^ Southeast Asia alone accounts for 90 million patients, of whom only half are aware of their diabetes.^2^ Five decades ago, type 2 diabetes mellitus was a rare disease, affecting just 1% of the population.^3^ Most people with type 1 diabetes had autoimmune insulin deficiency and would die within weeks to months without insulin therapy.^4^ How has this disease increased several fold, to affect 1 in 10 adults worldwide within a few decades? This is despite 10 Nobel Prizes being awarded over the past 100 years for diabetes-related scientific breakthroughs.^4^ With accumulating evidence for dietary interventions, more patients may prefer lifestyle modifications over gastric bypass surgery, for safe and effective diabetes remission.^5,6^ Plant-based whole food, low-calorie diet and even gastric bypass surgery require holistic lifestyle changes to sustain the desired weight reduction. Are we focusing medical resources on management rather than remission or cure?^7^ Can we identify which individuals with diabetes will benefit most from holistic interventions?

## Origins of type 2 diabetes

Excessive food consumption by the affluent has been directly linked to diabetes for thousands of years.^8^ In the 1930s when the prevalence of diabetes was <1%, only a fraction of these patients did not adequately respond to insulin injection and were considered insulin ‘resistant’. This observation opened the door to diagnosing type 2 diabetes. Since then, diabetes has been widely recognized as a chronic disease that occurs either when the pancreas does not produce enough insulin (type 1) or when the body cannot effectively use the insulin it produces (type 2 or ‘insulin resistant’).^9^ Our group has a strong interest in reframing this basic tenet and recently reviewed the evolution of the term ‘insulin resistance’.^10^ In later stages of the disease, all patients with diabetes manifest excess fatigue, urination and thirst as a result of insulin deficiency. In addition, long-term vascular complications and cardiac risk that accrue over decades are identical in both type 1 and type 2 diabetes. Thus, in 1930, with limited understanding of decades-l ong prediabetes, it made sense to place all the hyperglycaemic conditions under the ‘diabetes’ umbrella.

Type 1 diabetes is a true endocrine deficiency of a specific hormone – insulin – with specific symptoms and acute illness, centred around high glucose levels. On the other hand, type 2 diabetes is often due to relative overnutrition, with normal endocrine function and variable insulin levels. Homeostatic mechanisms are often intact for decades and attempt to compensate for the excess energy consumption from calorie-rich foods and drinks.^6^ Nature’s innate intelligence engages adaptive mechanisms to store lipids, causing weight gain over time. Insulin resistance is often secondary, variable and occurs only as cellular glucose uptake potential is saturated. Insulin injection may not elicit a sufficient reduction in blood glucose levels near the innate endocrine adaptive threshold. We recognize that there is significant individual variation in insulin sensitivity based on several genetic, environmental and age-related factors. However, recent diagnosis of type 2 diabetes, younger age (below 60 years), a diet high in calories and processed food, and severe obesity (body mass index [BMI] >35 kg/m^2^) may identify reversible beta cell dysfunction secondary to obesity.^11^

**Table 1:** Overview of challenges when applying the type 1 diabetes criteria to type 2 diabetes and those with overweight hyperglycaemia (OH). Current severe obesity-related differences and health implications unique to OH are highlighted

	**Type I diabetes**	**OH (BMI >35 kg/m^2^ and diabetes**)	**Type 2 diabetes**
**Reasons for 1930s nomenclature**		
Hyperglycaemia	Yes	Yes	Yes
Symptoms of excess urination, thirst, weight loss and fatigue	Present	Asymptomatic for years to decades	Variable
Prevalence until 1960s	1% of adults, due to insulin deficiency	Rare	<0.25% of adults, typically in older individuals with obesity
**Evolving understanding of overweight hyperglycaemia**		
Aetiology, insulin levels	Primary autoimmune endocrine disorder with insulin deficiency	Primarily a lifestyle issue; excess endogenous insulin secretion for decades	Several genetic and environmental factors contribute, variable insulin levels
Current prevalence	<5–10%	30–35% of people with type 2 diabetes	>95% of all people with diabetes
Biological adaptive responses	Inadequate; thus, uniformly fatal within weeks to months if not treated	Intact; relative energy excess with weight gain for decades	Variable; declines with duration of diabetes, comorbidities and age
Symptom onset	At diagnosis	Delayed by decades	Delayed by years
Management	Medication	Lifestyle and diet changes	Diet, lifestyle and medication
Significant (>20%) weight loss	Need insulin therapy	Often may be able to discontinue all medication	Significant reduction in medication requirement
Prognosis	Lifetime disease management	High remission and cure potential	Variable
HALE impact with optimal management	Limited HALE impact	Significant HALE gains and cost savings	Modest HALE improvement
Healthcare expenses	Inevitable	Significant reduction following weight loss	Modest reduction following weight loss

*BMI = body mass index; HALE = healthy life expectancy; OH = overweight hyperglycaemia.*

## Overweight hyperglycaemia

Much of the world struggled for sufficient food until the end of World War II. Cyclical food scarcity was the norm and allowed utilization of stored fat reserves periodically. Thus, in 1948, type 2 diabetes was uncommon and most diabetes was type 1, and required insulin.^12^ The secure, abundant supply of food that we have experienced continuously since the 1950s in the West, and within the last 2–3 decades in many developing countries in Asia and Africa, is unprecedented in human evolutionary history.^13^ Type 2 diabetes has increased tenfold in the past five decades, tripling within 8 years in some populations, and now accounts for 95% of all diabetes worldwide.^3,14,15^ Extrapolating diagnostic thresholds from type 1 diabetes may not be ideal for individuals with type 2 diabetes who do not manifest symptoms for decades (*Table 1*). Targeted interventions to address lifestyle have not kept pace with sweeping changes in the workplace and global societal trends. Severe obesity (BMI >35 kg/m^2^) increased from 4% in 1980 to 13% in 2004, and accounts for half of all new diabetes cases in the USA.^16^ With severe obesity likely to reach 25% by 2030, the excess diabetes burden will affect especially women, black people and low-i ncome groups.^17^ There could be significant health benefits for labelling type 2 diabetes in younger patients with severe obesity as what it really is – overweight hyperglycaemia (OH).

OH is the overwhelming of the natural adaptive mechanisms, due to continued nutritional overload often encountered in sedentary individuals with severe obesity and recent-onset hyperglycaemia. With intense lifestyle interventions targeting ideal body weight, this entity can be reversed, especially in younger people below 60 years of age.

## Reframing the lifestyle issue

**Figure 1:**
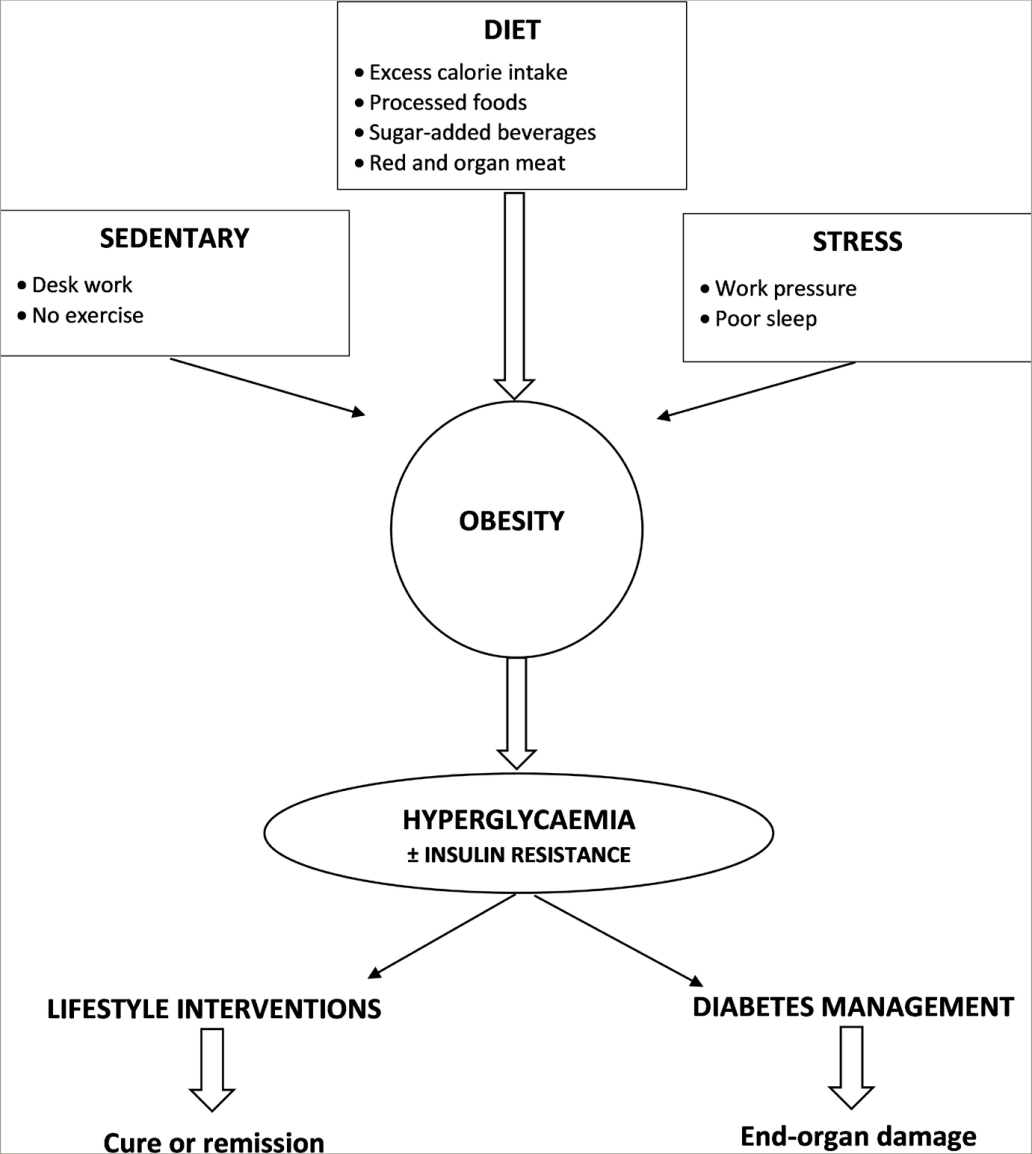
Outline of overweight hyperglycaemia aetiology, the secondary phenomenon of insulin resistance and the potential of lifestyle interventions to achieve remission

Medicine is advancing in great strides, with numerous new therapies addressing specific molecular, genetic and viral diseases. Type 1 diabetes management has benefitted from the pioneering work of several scientists, and with evolving continuous monitoring devices, long-term outcomes are likely to keep improving. The same cannot be said for type 2 diabetes. Thus far, we have used these same diagnostic criteria for OH. The longterm cardiovascular risk is a continuum without a clear demarcation to justify prediabetes as a separate entity. Based on individual genetic and environmental factors, some people manifest metabolic syndrome characteristics, while others manifest hyperglycaemia in the diabetes range. Use of the term OH will help to shift the focus onto the lifestyle and societal transformations needed to regain health. *Figure 1* outlines the framework of OH, the inconsequential role of insulin resistance, and the opportunity for lifestyle interventions aimed at remission/cure, rather than pharmacological chronic diabetes management and progressive end-organ damage and vascular complications.

## Biological resilience

Clinicians have the opportunity and responsibility to educate individuals about the significant developments in the fields of ageing and resilience. Calorie restriction in all normal-weight, healthy animal species increases survival by 20–200% beyond usual life expectancy.^18^ Calorie restriction in rhesus monkeys, who share 93% of their DNA with humans, dramatically reduces the risk of diabetes and metabolic syndrome.^19^ Human resilience starts to decline by the age of 40 years.^20^ Until about the age of 70 years, we may be able to engage anti-ageing and cellular resilience pathways to reverse diabetes.^20^ Normal-weight, healthy volunteers with a BMI of 25 kg/m^2^ demonstrated improved metabolic health through 20% calorie restriction.^21^ Insulin sensitivity and cardiometabolic risk appears optimal at a BMI of 22 kg/m^2^ for healthy humans.^21^ With life expectancy increasing globally, these studies suggest we may increase healthy life expectancy (HALE) by lowering daily calorie intake recommendations.^22^

## Gastric bypass and fasting

Younger people with severe obesity may lose 10 years of life expectancy.^23^ Gastric bypass surgery improves diabetes control, yet the long-term physical, nutritional and psychological risks need further evaluation.^24^ When performed relatively early, between the ages of 30 and 50 years, bariatric surgery restores 3 years of life.^25^ Frailty may contribute to the significant drop in gastric bypass procedures in older patients, with surgery rates in younger people fortyfold higher than in those over the age of 65 years.^26^ Chronic conditions such as heart disease further reduce the suitability for bariatric surgery.^27,28^

'Siddha', a traditional Tamil medicine used for several thousand years, recommends two meals a day for maintaining good health, and prescribes further restriction to only one meal a day to aid healing. Unfortunately, especially within the past few decades, average meal frequency has increased to 5–6 meals a day.^29^ Autophagy research from Japan recently reaffirmed the therapeutic potential of fasting, with Dr Yoshinori Ohsumi being awarded the 2016 Nobel Prize in Physiology or Medicine.^30–32^

Our group encourages cardiac patients with cardiometabolic disorders to explore the Siddha mindful eating practice called ‘Hunger Gratitude Experience’, which combines mindfulness, gratitude, active relaxation and fasting to sustain holistic weight loss in the long term.^33^ When cardiac patients lose 20–35% of body weight, they are often able to discontinue several diabetes medications.^34^ Independently of weight loss, the natural ketosis induced by fasting may result in pleiotropic metabolic, cardiac and cognitive benefits.^35^

## Insulin resistance

Insulin deficiency and resistance are considered the central issues in type 2 diabetes, with novel pharmacological agents potentially addressing a dozen disease-specific targets.^36^ However, insulin levels are often adequate in OH. Humans have evolved over millions of years to expect food scarcity several times in a lifetime. A constant supply of food is indeed a recent ‘problem of plenty’. In clinical practice, insulin levels and insulin resistance are seldom measured, and do not add value in severe obesity. Abnormal weight gain may occur within ‘normal’ BMI range based on individual race, stress, activity level, age and body morphology. The global diabetes epidemic does not affect primitive tribes and people continuing to live traditionally in regions of the world with a high number of centenarians and elderly people without disease or chronic health problems.^37^ All people with type 2 diabetes have insulin resistance, which renders insulin testing redundant in patients with severe obesity.^38^ Several biological and hormonal factors have been linked to the increasing global prevalence of metabolic syndrome.^39^

Interestingly, insulin resistance resolves completely with 30% calorie restriction in rhesus monkeys.^19^ Emerging evidence for remission places obesity, instead of insulin resistance, at the centre of the type 2 diabetes paradigm.^40^ Healthcare would improve if providers shifted the focus from insulin resistance concerns to lifestyle modification and weight reduction. Redirecting resources first towards addressing lifestyle changes before exploring genetic testing and molecular therapies may better address global health equity.

## Shooting the messenger

OH, like prediabetes, remains asymptomatic because of adaptive weight gain over decades. Therefore, unlike type 1 diabetes where patients develop severe hyperglycaemia and catabolic symptoms, individuals with OH may appear well with no symptoms for many years. Often organ damage with cardiac or renal failure brings OH to medical attention. If there are no symptoms and patients can reverse to normal metabolism, then the hyperglycaemia of type 2 diabetes may just be an epiphenomenon. We diagnose cardiac, hepatic or renal pathology based on troponin elevation, hyperbilirubinemia and uremia, respectively. We do not attempt to reduce or ‘control’ these serum biomarker levels. Instead, we identify and manage the underlying aetiology of organ injury to reduce long-term damage. OH manifests hyperglycaemia as a disease marker decades before normal adaptive mechanisms are overwhelmed. The underlying pathology needing management is sedentary lifestyle, mental stress, unhealthy and inappropriate diet, and overnutrition. Therapies focusing on controlling hyperglycaemia are shooting the messenger without addressing the underlying problem, and are unlikely to improve outcomes. We miss the window of opportunity to address the social, nutritional and lifestyle problems, and thereby fail to correct the underlying relative overnutrition.

## Management and outcomes

Lifestyle changes are recommended for people with prediabetes to prevent diabetes. This is welcome and central for public health. However, people with OH are considered in the ‘chronic disease’ category, even though a majority would benefit significantly from changes to lifestyle.^41–44^ Insulin as well as oral antidiabetic agents reduce blood glucose levels but fail to reverse the disease or improve survival. Gastric bypass reverses OH, giving us the evidence we need that this is indeed a metabolic problem.^45^ Importantly, lifestyle interventions may offer all the metabolic benefits of gastric bypass with adequate calorie-restricted weight reduction.^46^ Many patients with long-standing diabetes showed reversal of metabolic abnormalities and could discontinue diabetic medications following a 20% weight reduction.^40^

## Low-income countries

Low-i ncome Southeast Asian, African and Latin American countries have limited resources to manage diabetes. Much of the developing world uses Western standards without validating ‘normal’ ranges for cardiometabolic risk predictors. Developing countries may not have developed the genetic tolerance to overnutrition, developing diabetes decades earlier and at lower BMI compared with Western populations.^47^ Thus, a BMI of >23 kg/m^2^ may represent overweight and >25 kg/m^2^ may denote obesity, causing diabetes and other metabolic derangements.^48^ Limited resources and challenges accessing therapies in developing countries often result in premature cardiovascular mortality.^49^ Thus, it is vital to deliver appropriate education to providers and patients about low-cost, sustainable lifestyle and dietary interventions to optimize metabolic health in low-i ncome countries.

## Future directions

OH is a lifestyle disease with some genetic predisposition. The pace of modern life, competition and mental stress are likely to contribute significantly to sympathetic activation and insulin resistance. Furthermore, mental stress may predispose to unhealthy ‘comfort’ food choices and ‘emotional’ eating behaviour patterns with cumulative risk over a lifetime. The role of social media in adding and aiding stress and influencing behaviour also needs closer scrutiny.

Mind–body methods that can address the root causes of OH in sustainable and meaningful ways are required. Integrative approaches that incorporate local cultural fasting traditions may offer solutions. Appropriate food (diet) is central to achieving meaningful weight loss. Research into sustainable lifestyle changes, diet and physical activity are needed to improve HALE, minimize frailty and optimize compliance.^50^ OH may contribute to the USA and India having the lowest HALE within their income categories.^51^

Public health policy and corporate workspace design should account for the basic human requirement of physical activity and movement. Improving OH requires the major health challenge of obesity and mental stress to be addressed. Real solutions may be years away, but first we must acknowledge that the problem has burgeoned well beyond the scope of medical solutions. Labelling diabetes with severe obesity as OH may allow a broader societal reckoning. Individuals can and should be identified as early as possible, and should be fully informed that OH is much more likely to be due to overnutrition than insulin resistance or a genetic ‘defect’. For the 35% of people with type 2 diabetes and OH, and the 34% of all adults dealing with prediabetic conditions globally, it is crucial to emphasize that they can regain their health by reducing weight to a targeted ideal body weight. Calorie counting, exercise time and glycosylated haemoglobin are only intermediate-term goals. Reaching ideal body weight will soon become a sustainable reality with advances in lifestyle medicine. Then, the persistence of hyperglycaemia after achieving normal body weight will be needed to diagnose type 2 diabetes.

## Conclusions

We have not made meaningful progress in addressing type 2 diabetes since its first description in the 1930s. Nature is better adapted to cope with undernutrition than overnutrition. This fact is painfully felt as metabolic disorders surge just as human life expectancy is increasing. This is an opportune moment to realize that the latest scientific discoveries cannot overcome sedentary habits and overnutrition. We may find solutions for this when we reframe the entity as a lifestyle issue. While this may seem to ‘state the obvious’, healthcare providers have the responsibility to present to society the daunting issues around obesity and metabolic disorders. Low-i ncome countries and the underprivileged are dealing with food insecurity and obesity simultaneously. The challenges are region-specific and complex, but innovative social and lifestyle solutions will emerge. Biology is innately intelligent. When well informed, people with OH may find intuitive lifestyle solutions to reduce food consumption, eat healthily and increase their activity levels, rather than accepting the inevitable progression of diabetes and/or waiting for the next miracle cure pill for type 2 diabetes to be discovered.

## Learning points

- 1. Doctors must recognize the curative potential in OH in younger people with diabetes and severe obesity.
- 2. Doctors must recommend lifestyle and food modification for substantial weight reduction.
- 3. Lifestyle changes in younger people engages innate resilience to reverse OH.
- 4. HALE may improve, particularly for the USA and India, if we address OH.
